# Autophagy regulates endosymbiont distribution in early *Drosophila* embryogenesis

**DOI:** 10.1080/27694127.2022.2112124

**Published:** 2022-08-12

**Authors:** Anton Strunov, Katy Schmidt, Martin Kapun, Wolfgang J. Miller

**Affiliations:** a Department of Cell and Developmental Biology, Medical University of Vienna, Schwarzspanierstrasse 17, 1090, Vienna, Austria; b Central Research Laboratories, Natural History Museum of Vienna, 1010 Vienna, Austria

The majority of animals on our planet engage in symbioses with microbes. The success of a microbial symbiosis depends on the contribution of both partners and their ability to find a cost-benefit equilibrium where everyone profits and propagates further. In stable host-microbe associations, the host usually controls symbiont titer and tropism to alleviate the potentially high costs of infection, whereas vertically transmitted microbes favor the host´s fitness to guarantee their own transmission into the next generation. Understanding this complex interplay between partners and deciphering the mechanisms that maintain homeostasis is fundamentally important and may be applied in fighting human-relevant infectious diseases like malaria or Dengue fever.

In the well-studied symbiosis between *Wolbachia* bacteria and *Drosophila* flies, the intracellular microbe is maternally transmitted and colonizes the female gonads to propagate in the next generation. Any somatic localization, however, is a dead end for *Wolbachia* and might lead to decreased host fitness when titers are costly. The range of *Wolbachia* tissue infection varies from systemic (in highly permissive *Drosophila* species such as *D. melanogaster*), to restrictive, such as in some neotropical *Drosophila* species which bear isolated infection clusters in specific tissues. In the latter, the host is apparently unable to tolerate high densities of bacteria due to their overall deleterious effects and hence must regulate their number by selective tissue tropism.

**Figure 1.**
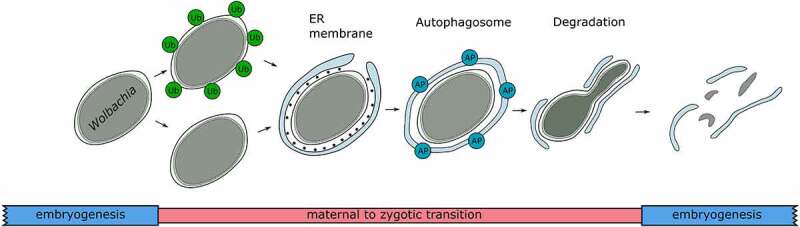
Schematic representation of *Wolbachia* elimination from the soma of neotropical *Drosophila* flies. Early embryos show systemic infections, but active *Wolbachia* clearance starts at the maternal-to-zygotic transition stage of embryogenesis with the recognition of bacteria through ubiquitin-dependent (Ub; green), or Ub-independent mechanisms, followed by engulfment by the endoplasmic reticulum (ER) membrane (light blue). Next, autophagosomes (APs; blue) are formed for degradation via twisting and stretching the bacterial cell, the “bondage” phenotype, until complete elimination. Modified from [1].

In our recent publication [1] we uncovered the mechanism of *Wolbachia* restriction in neotropical *Drosophila* hosts. First, we investigated the localization of bacteria in the soma and the gonads of six *Drosophila* species at different stages of development to identify the exact moment of restriction. Most neotropical species show similar restriction patterns at larval and adult stages. Experiments at successive developmental stages revealed that early embryos are systemically infected with *Wolbachia*, but during the maternal-to-zygotic transition (MZT), when the embryo starts transcription autonomously, bacteria vanish from most embryonic cells, except for the primordial germ cells (PGCs), the future gonads of the fly and a few defined neuroblasts (Figure 1). In *D. melanogaster* and mosquitos, autophagy was previously shown as the key regulator of *Wolbachia* titer in adults. Using an antibody against the autophagic marker Atg8a we now demonstrate that the same cellular clearance mechanism eliminates *Wolbachia* from embryonic tissues during MZT. Interestingly, closely related neotropical species exhibit both ubiquitin-dependent and ubiquitin-independent autophagy pathways, demonstrating their plasticity, which might be host dependent (Figure 1). In contrast, autophagy is absent in PGCs, which makes this cell type a safe haven for the endosymbiont and provides a secure route via the female gonad to the future progeny. Ultrastructural analysis revealed the active role of endoplasmic reticulum (ER) membranes in the elimination process. During the MZT, membranes of the ER encircle *Wolbachia* cells and attract the autophagy machinery for the active degradation of bacteria (Figure 1). Surprisingly, no bacteria lysis in autophagic vacuoles is observed, but extremely elongated and crooked shapes are found instead. These data show a novel and unconventional mechanism of bacterial degradation and clearance in the host cells, which resembles the previously described elimination process of plastids in *Brassica* plants.

We uncovered a unique system of controlling bacterial titer and tropism through ER-mediated autophagy at the early embryonic stage when the embryo takes over the transcriptional control. While we were able to dissect the steps of bacterial elimination in detail, we still do not understand how this process retains the bacteria in PGCs and degrades them in the soma. Given that two distant neotropical *Drosophila* subgroups, *sturtevanti* and *willistoni*, developed similar mechanisms of infection restriction, the elimination process must be conserved and very efficient. One hypothesis is that residing in the PGCs is a refugium for *Wolbachia*. This type of cell is indeed transcriptionally quiescent in early embryogenesis and, hence, autophagy might be inactive, providing an essential opportunity for the microbe to settle. Alternatively, bacteria themselves might be able to avoid autophagy in the PGCs by modulating their surface markers and hiding from the recognition system of the cell. The elimination of bacteria coincides with the massive removal of maternal mRNAs and activation of zygotic gene expression during the MZT, suggesting that *Wolbachia* degradation might result from a nonspecific antibacterial immune response of the embryo. Modulating the outer membrane markers of the endosymbiont might work as an invisibility cloak in PGCs and in a subset of somatic stem cells, such as neuroblasts. Bacterial effector proteins are major players in modulating microbe-directed interactions with the host cell. As recently uncovered in PGCs of *D. melanogaster*, the *Wolbachia* protein TomO interacts with different mRNA molecules, elevates the expression of *Nanos* mRNA, and thereby supports the host’s reproductive success. We hypothesize that, during the MZT, TomO or other unknown *Wolbachia* effector proteins might enable this endosymbiont to escape the autophagy machinery in PGCs. Similar mechanisms might be also involved in the survival of *Wolbachia* in *Drosophila* species with no restriction patterns. To dissect the major players of a host-symbiont association and to decipher the exact mechanism of bacterial recognition, targeting, and elimination, it is crucial to further study the effector proteins of *Wolbachia* and their targets in different *Drosophila* species.
